# Viral manipulation of vector behaviour: cucumber mosaic virus has differential effects on specialist *versus* generalist aphids infesting *Arabidopsis thaliana*

**DOI:** 10.1186/s12985-026-03081-w

**Published:** 2026-01-25

**Authors:** Hana Azuma, Alex M. Murphy, Nik J. Cunniffe, Arden G. Berlinger, John P. Carr

**Affiliations:** Department of Plant Sciences, University of Cambridge, Cambridge, CB2 3EA UK

**Keywords:** Attract-and-Deter, Virally induced plant phenotype, Latent period, Non-persistent transmission, Neural-Constraints, Sequential-Cues, Viral suppressor of RNA silencing

## Abstract

**Background:**

Cucumber mosaic virus (CMV) is vectored by aphids. Infection of *Arabidopsis thaliana* plants with CMV affects their attractiveness to aphids (*Myzus persicae*) and the performance of aphids confined on these plants. CMV-induced changes in plant-aphid interactions (‘viral manipulation’) may promote transmission. *M. persicae*, an efficient CMV vector is a ‘generalist’, i.e., it has many plant hosts. *A. thaliana* is also exploited by crucifer-specialist aphids including *Lipaphis erysimi* (an efficient CMV vector) and *Brevicoryne brassicae* (a poor CMV vector). We explored the hypothesis that CMV-induced viral manipulation of aphid behaviour would exert stronger effects on *M. persicae* than on crucifer-specialists.

**Results:**

*M. persicae*, *B. brassicae* and *L. erysimi* were released in microcosms and allowed to choose to settle on either CMV-infected or mock-inoculated plants. Initial experiments showed that as systemic CMV infection developed in *A. thaliana*, aphids of *M. persicae* were decreasingly likely to settle on infected plants. In subsequent experiments, using plants at 14 days post-infection, it was found that aphids of *M. persicae* were faster to choose between infected and uninfected plants than specialist aphids, but that both the generalist and specialists were less likely to settle on CMV-infected plants. Olfactometry showed that volatiles emitted by CMV-infected plants attracted *M. persicae*, and although the specialists showed no significant preferences, greater numbers of aphids of all three species responded when CMV-infected plant volatiles were presented to them.

**Conclusions:**

As CMV infection develops, *A. thaliana* becomes less susceptible to aphid colonisation, however, plants continue to emit attractive olfactory cues. This is consistent with a model in which aphids are attracted to infected plants but discouraged from settling (e.g., by gustatory cues), which encourages aphids to carry CMV to non-infected plants. CMV appears to be more successful in manipulating the interactions of *A. thaliana* with the generalist aphid *M. persicae*, than with the crucifer specialists *B. brassicae* or *L. erysimi*.

**Supplementary Information:**

The online version contains supplementary material available at 10.1186/s12985-026-03081-w.

## Background

Cucumber mosaic virus (CMV) is a positive-sense RNA virus with a tripartite genome that encodes five proteins [1]. Taxonomically the virus is classified as the species *Cucumovirus CMV* in the Genus *Cucumovirus* in the Family *Bromoviridae* [2]. CMV has many strains, and most can be placed into Subgroups IA, IB and II, according to RNA sequence data [3]. The Fny-CMV strain [4] is among the best studied and can infect a wide range of plants including the model plant *Arabidopsis thaliana*, which is also a host for CMV under natural conditions [5].

CMV is vectored by aphids in the non-persistent mode [6]. This means that CMV virions are retained for only minutes to a few hours in the tip region (acrostyle) of an aphid’s stylet [7–9]. CMV virions are acquired from the epidermal cells of infected plants within a few seconds of ingestion and are released rapidly during salivation when the aphid probes the epidermis of another plant [10]. The loose binding of CMV virions to the acrostyle is mediated by interactions between the viral coat protein [11] and insect cuticular proteins [7–9]. Other viral proteins exert indirect effects on CMV transmission by modifying the host plant phenotype in a manner that may influence plant-aphid interactions to alter virus transmission dynamics [12–14]. One such ‘virally modified plant phenotype’, dubbed ‘attract-and-deter’ [15] was first reported in squash (*Cucurbita pepo*) plants infected with Fny-CMV [16]. These infected plants emitted a blend of volatile organic compounds (VOCs) that attracted the polyphagous aphids *Myzus persicae* and *Aphis gossypii*, but these herbivorous insects were deterred from settling and prolonged feeding by a decrease in the palatability of the plant tissues [16]. Mathematical modelling indicated that this combination of attractive olfactory cues and deterrent gustatory cues will accelerate transmission by aphids of CMV or any other non-persistently transmitted viruses from an infected host to neighbouring uninfected plants [15, 17].

Studies of virally modified plant phenotypes using *A. thaliana* Col-0 and Fny-CMV showed that the virus induces resistance to *M. persicae* that is characterised by decreased phloem feeding and decreased growth and reproduction of aphids confined on infected plants [12]. The resistance to aphids which appears to form the basis of the CMV-induced deterrence to settling, involves at least two mechanisms that are both induced by the viral 2a protein, which among other things, is also the CMV RNA-dependent RNA polymerase [13, 14]. The two mechanisms underpinning resistance to *M. persicae* both depend upon signalling mediated by the phytohormone jasmonic acid but one inhibits aphid growth and requires activation of pattern-triggered immunity (PTI), while the other mechanism inhibits reproduction and is PTI-independent [14].

While *M. persicae* is a polyphagous herbivore that can feed on and colonise many plant species [18, 19] there are many aphid species specialising on specific plant groups. For example, *Lipaphis erysimi* and *Brevicoryne brassicae* have host ranges limited to the *Brassicaceae* (the crucifers), which is the plant family to which *A. thaliana* belongs. In contrast to what was first observed with *M. persicae* [12], growth of neither *L. erysimi* nor *B. brassicae* was inhibited when aphids of these two aphids were confined on CMV-infected *A. thaliana* plants, although *B. brassicae* reproduction was inhibited [14]. These data suggested that the ‘deter’ aspect of the CMV-induced ‘attract-and-deter’ host phenotype [15], is less effective at influencing crucifer specialist aphids than a generalist such as *M. persicae*. To improve understanding of virally modified plant phenotypes we investigated the role of VOCs in providing the ‘attract’ component of CMV-induced ‘attract-and-deter’ host phenotype in *A. thaliana*, if changes in VOC emission affected crucifer-specialist aphids, and how previously observed effects of CMV-induced resistance mechanisms on generalist versus specialist aphids [20] affected aphid settlement on infected plants. In this study we tested these hypotheses and addressed an important knowledge gap regarding how long it takes for the virally induced deterrence phenotype to manifest following infection with CMV, since this may have epidemiological importance.

## Methods

### Biological materials

*Arabidopsis thaliana* Heyn. Col-0 seed stocks were originally obtained from the Nottingham Arabidopsis Stock Centre. Plants to be used for viral infection and aphid studies were grown in a 4:1 compost/sand mixture at 21 °C in a custom-built growth room (Conviron, Manitoba, Canada) with a regime of 8 h of light and 16 h of darkness and light intensity of 200 µE.m^–2^.s^–1^ [21]. Preparation of apterous specialist and generalist aphids for experimentation has been described previously [14]. Clonal cultures of *Myzus persicae* Sulz. clone US1L [22], *Brevicoryne brassicae* L. and *Lipaphis erysimi* Kaltenb. [23], both kind gifts from Rothamsted Research, were propagated on plants of Chinese cabbage (*Brassica rapa* var. *pekinensis* ‘Green Rocket’: Tozer Seeds, Cobham, UK). Cucumber mosaic virus strain Fny [4] was reconstituted by agroinfection of *Nicotiana benthamiana* Domin with infectious cDNA clones for the three genomic RNAs [24, 25]. Virions of Fny-CMV were purified from systemically infected leaves of *N. benthamiana* as described by Palukaitis [26]. Virions diluted to 100 µg.ml^− 1^ in sterile water were mechanically inoculated onto Carborundum-dusted lower leaves of *A. thaliana* plants at the 4–6 leaf stage [21]. Mock inoculation used sterile water on Carborundum-dusted lower leaves. Successful infection of plants was confirmed using a double antibody sandwich enzyme-linked immunosorbent assay kit for CMV (Bioreba) and a Titertek Multiskan Plus microplate reader with DeltaSoft software.

### Aphid free choice assays

Aphid two-way free choice settling assays used wingless (apterous) adult *M. persicae* and *B. brassicae* and *L. erysimi* that were 7 days old (for all three species). To obtain these aphids for experimentation, 200–300 adult aphids were transferred with fine-tip paintbrushes to un-infested Chinese cabbage plants and allowed to reproduce for 24 h before removal. The nymphs produced during this period were allowed to develop for an additional 6 days before use. Groups of 10–15 aphids were placed inside 1.5 ml microcentrifuge tubes, and the tube was placed upright in compost equidistantly between plants that were 9 cm apart in pots contained in micro-perforated film (Associated Packaging Ltd., Tonbridge, Kent, UK) in mesh insect cages (Insect Cage Net, Carmarthen, Dyfed, UK) [20]. Apterous aphids were released by opening the tube lid and aphids were presented with choices between pairs of identically treated plants (i.e., infected versus infected, mock-inoculated versus mock-inoculated) or pairs of plants that were either infected with CMV, or which had been mock-inoculated. Aphid settlement on plants was recorded at 1 and/or 24 h post-release. In some experiments settlement assays were carried out with plants that had been inoculated or mock-inoculated 3, 9 or 21 days previously. In most experiments plants were used at 14 days post-inoculation (dpi) with CMV or following mock inoculation.

### Olfactometry

Two-way olfactometry using *M. persicae*, *L. erysimi* and *B. brassicae* utilised the apparatus described by Safari et al. [27], presenting aphids with choices between VOCs emitted by CMV-infected and mock-inoculated plants and controls in which the insects were presented with VOCs emitted by paired mock-inoculated plants or paired CMV-infected plants. Fifty 7-days-old aphids were used in each assay and the number of aphids that made a choice was recorded 24 h after release.

### Statistical analyses

**Fig. 1:**
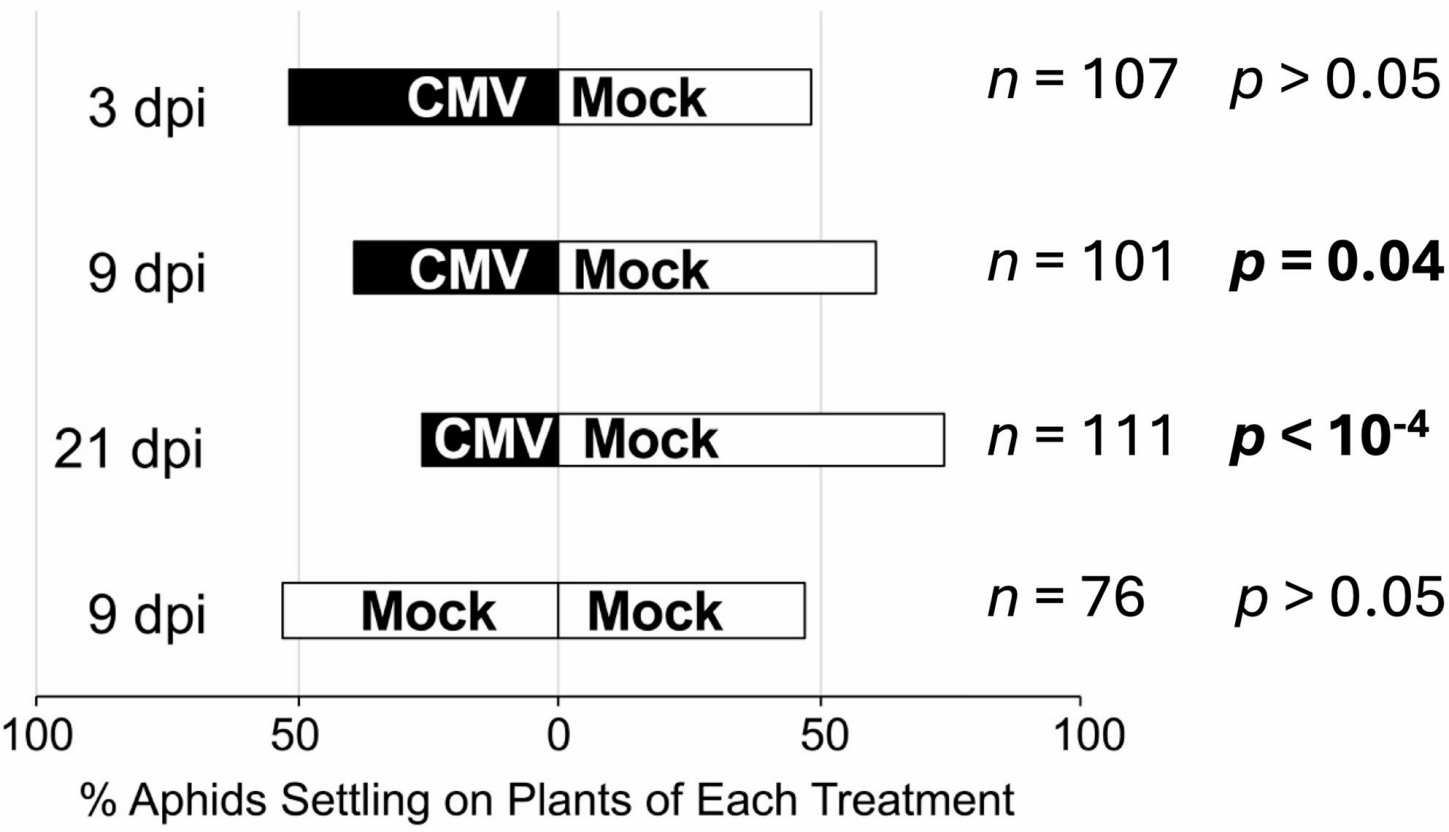
Changes in settling patterns for the aphid *Myzus persicae* on mock-inoculated plants and plants infected with cucumber mosaic virus over time. Two-way free choice settling assays were carried out using *Myzus persicae* to determine the preferences of aphids to settle on mock-inoculated *Arabidopsis thaliana* plants (Mock) versus plants infected with cucumber mosaic virus (CMV). Plants were inoculated on lower leaves and aphid settling assays carried out at 3-, 9- and 21-days post-inoculation (dpi) with settled aphids counted at 24 h post-release. In total 120 aphids were released per experiment per timepoint (12 groups of 10 aphids). Control assays (8 groups of 10 aphids) used only mock-inoculated plants and settling was assessed at 9 days following mock inoculation on lower leaves. Differences in settling behaviour at each timepoint were assessed for statistical significance (*p* < 0.05: in bold) using a Wald test on the relevant fixed effect coefficient of a binomial generalised linear mixed model, fitted without an overall intercept term

**Fig. 2:**
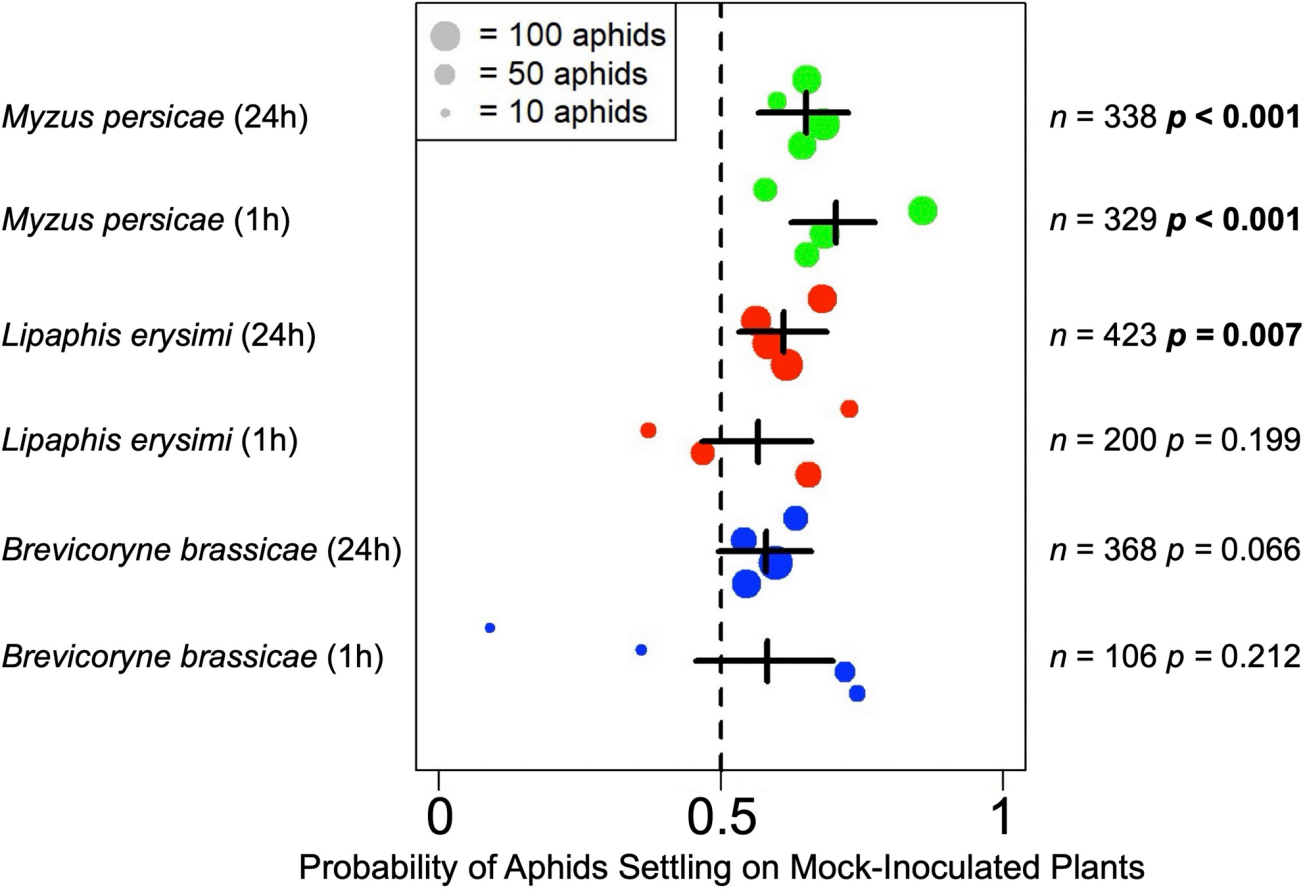
Summary of aphid preferences to settle on plants that had been mock-inoculated or infected with cucumber mosaic virus. Two-way free choice aphid settling assays were carried out using *Myzus persicae* (generalist), *Lipaphis erysimi* and *Brevicoryne brassicae* (both crucifer specialists). Aphids were released in arenas with equidistantly placed *Arabidopsis thaliana* plants that had been mock-inoculated or infected with cucumber mosaic virus 14 days previously and the numbers of aphids of each species that had settled on plants (*n*) were recorded at 1 and 24 h post-release. The total number of aphids that were released for each species across the experiments was 435 (*M. persicae*), 440 (*L. erysimi*), and 470 (*B. brassicae*). This figure summarises the results of these free choice settling experiments (File S1; Figure S1) to determine the probability (odds-ratio) of aphids of each species choosing to settle on mock-inoculated plants in settling assays. The vertical dashed line at 0.5 indicates that the probability of aphids choosing either treatment was 50%, i.e. no significant preference. Each dot represents an independent experiment with the probability of settling on mock-inoculated plants for the aphids *M. persicae* (green), *L. erysimi* (red) and blue = *B. brassicae* (blue). The statistical significance (*p* < 0.05) of any preference was assessed using a Wald test on the relevant fixed effect coefficient of a binomial generalised linear mixed model, fitted without an overall intercept term and with individual experiments (i.e., dots) as a random effect (significant values are indicated in bold type). The horizontal black bars span the 95% confidence interval for aphids of each species choosing to select at each time

**Fig. 3:**
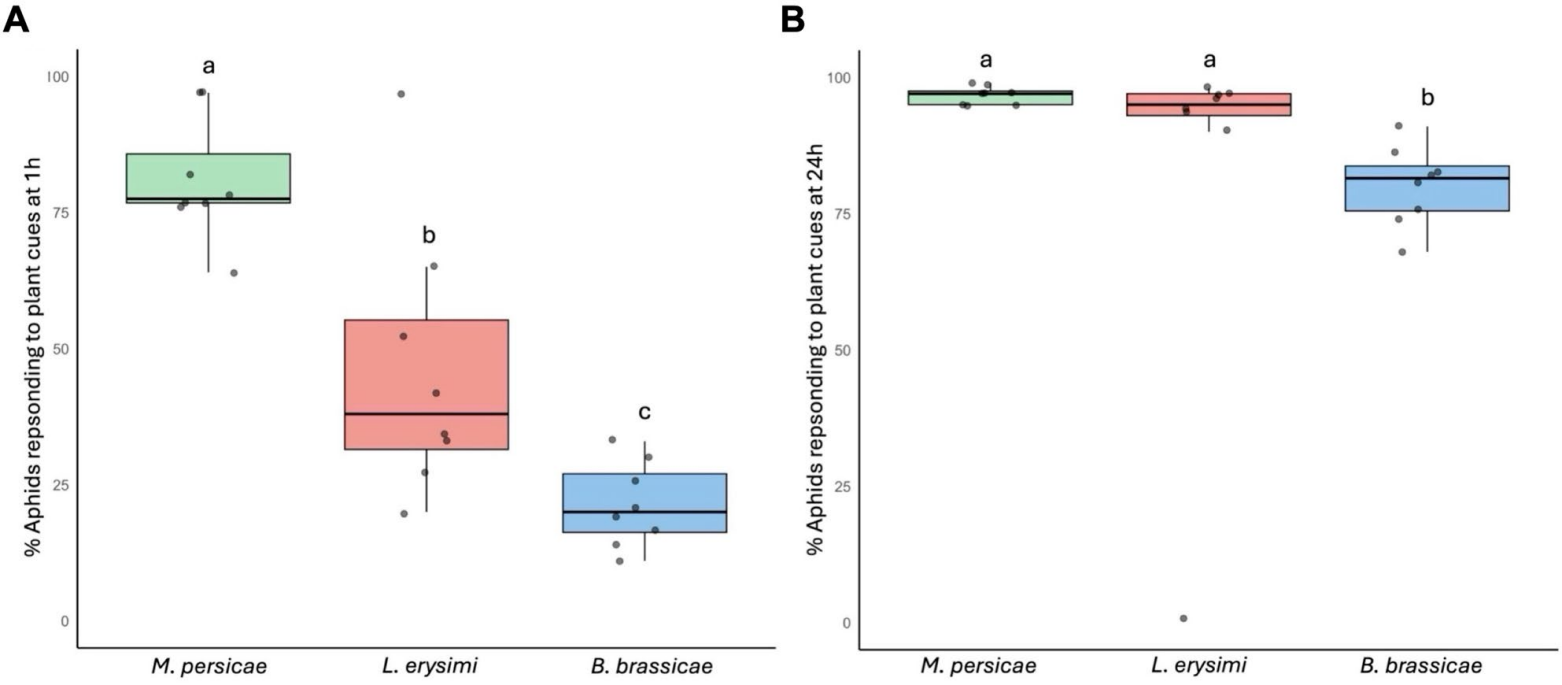
Differences between a generalist and two crucifer specialist aphids in their tendency to settle on plants. The data from the two-way free choice settling assays (Fig. 2; File S1; Figure S1) was analysed to identify consistent differences in the tendency of the generalist aphid *Myzus persicae*, and the two crucifer specialists, *Lipaphis erysimi* and *Brevicoryne brassicae*, to migrate towards and settle on *Arabidopsis thaliana* plants, regardless of infection status. At 1 h post-release (**A**) *M. persicae* typically showed a settlement rate of around 80% (of 685 aphids tested), while the respective settlement rates at this time-point for *L. erysimi* (710 aphids tested) and *B. brassicae* (780 aphids tested) were around 45 and 20%. By 24 h post-release > 95% of *M. persicae* and *L. erysimi* had settled on plants; rates that were still significantly higher than the rate for *B. brassicae* (*c*. 80%) at this time point (**B**). Each dot represents the percentage of aphids settling on plants (irrespective of plant infection status) in a single experiment. Differences in settling behaviour at each time point were analysed by binomial generalised linear mixed models, fitted with individual experiments (i.e., dots) as a random effect, and using *post-hoc* tests for pairwise comparisons between treatments (i.e., aphid species), with values that were significantly different (*p* < 0.05) indicated by different lower-case letters

**Fig. 4:**
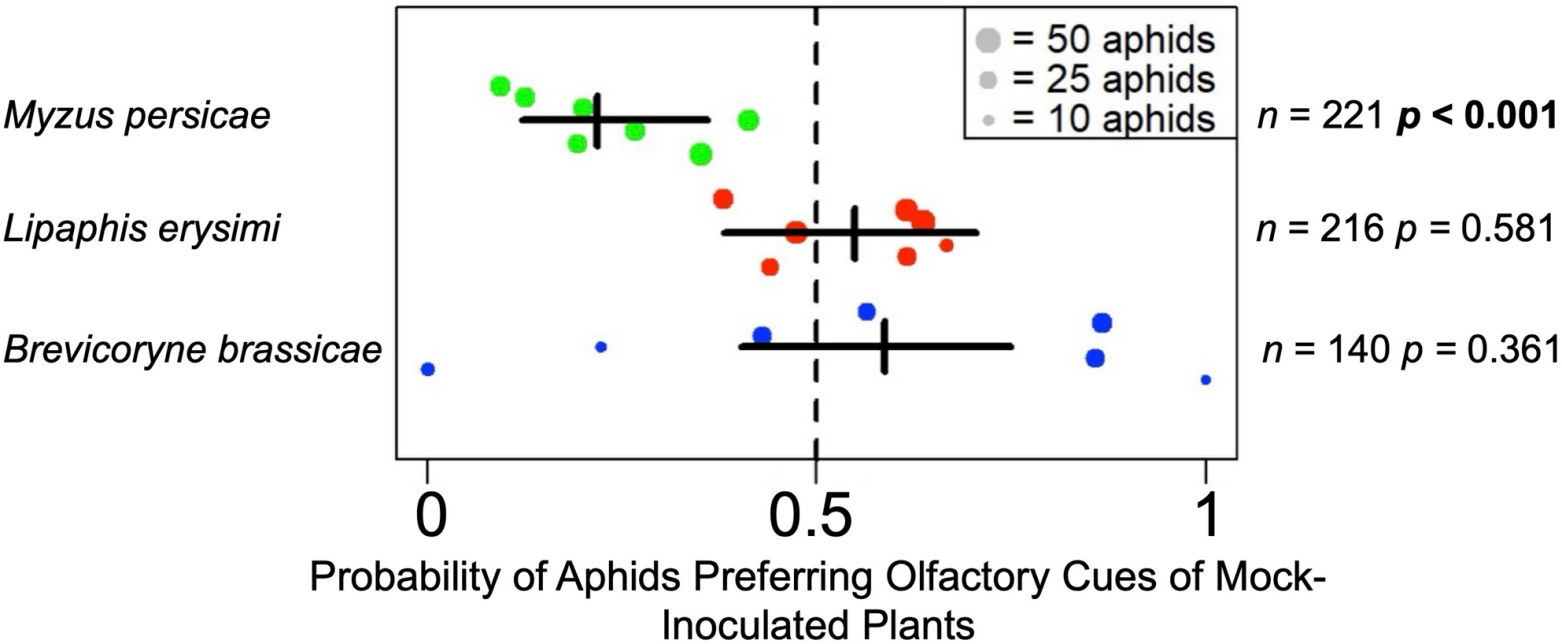
Summary of olfactometry analyses to determine generalist and crucifer-specialist aphid preferences for volatile organic compounds emitted by mock-inoculated versus plants infected with cucumber mosaic virus. Olfactometry was used to investigate the preferences of *Myzus persicae* (generalist), *Lipaphis erysimi* and *Brevicoryne brassicae* (both crucifer specialists) for volatile cues emitted by *Arabidopsis thaliana* plants mock-inoculated or infected with cucumber mosaic virus 14 days previously. Choices were recorded at 24 h post-release. Fifty aphids were investigated in each olfactometer assay and experiment were carried out seven times for each species, and the results used to determine the probability (odds-ratio) of aphids of each species showing a preference to move towards the olfactory cues presented by mock-inoculated plants in olfactometry assays. Each dot represents an independent experiment with the probability of settling on mock-inoculated plants for the aphids *Myzus persicae* (green), *Lipaphis erysimi* (red) and *Brevicoryne brassicae* (blue). The number (*n*) of aphids of each species that made a choice across all experiments is indicated, in each of 7 replicates the number of aphids released was 50, i.e., the total was 350 aphids for each species. The vertical dashed line at 0.5 indicates that the probability of aphids choosing either treatment was 50%, i.e. no significant preference. The statistical significance (*p* < 0.05) of any preference was assessed using a Wald test on the relevant fixed effect coefficient of a binomial generalised linear mixed model, fitted without an overall intercept term and with individual experiments (i.e., dots) as a random effect (significant values are indicated in bold type). The horizontal black bars span the 95% confidence interval for aphids of each species choosing to select either treatment

**Fig. 5:**
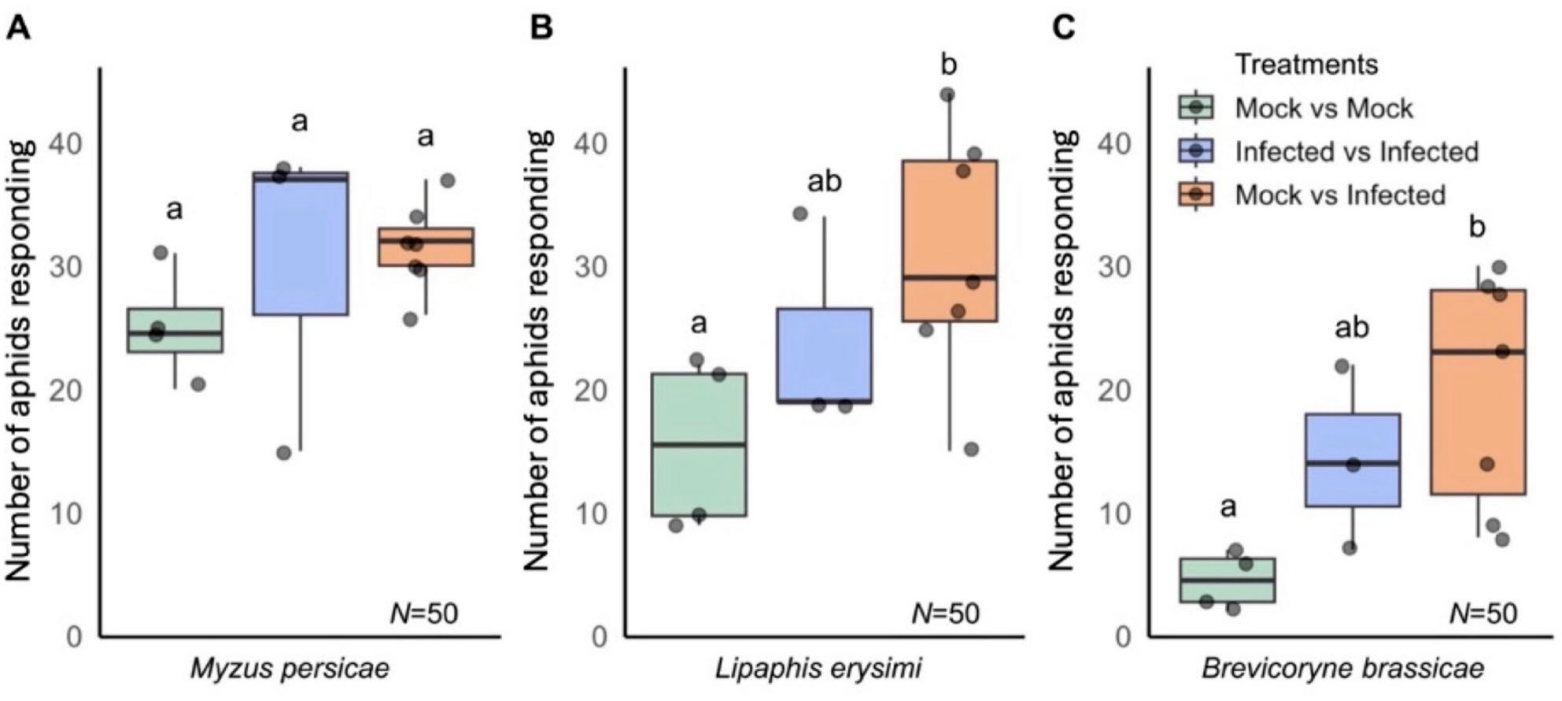
Generalist and specialist aphid responses to *Arabidopsis thaliana* olfactory cues. Data from olfactometry assays (Fig. 4; File S1) was used to determine if aphids were more or less likely to choose to move towards any source of plant volatile organic compounds (VOCs) in the presence of VOCs from CMV-infected plants, those emitted by mock-inoculated plants (Mock), or when both VOC blends were present in the olfactometer. Responses of the generalist aphid *Myzus persicae* (**A**), and the crucifer specialists *Lipaphis erysimi* (**B**) and *Brevicoryne brassicae* (**C**) were compared. Each dot represents an independent experiment with the mean number of aphids responding (regardless of whether the choice was for VOCs emitted by CMV-infected versus mock-inoculated plants) in each experiment indicated on the Y-axis. In each experiment the total number of aphids released per treatment (*N*) was 50. There were 4 replicate experiments for Mock vs. Mock, 3 replicate experiments for Infected vs. Infected and 7 replicate experiments for Mock vs. Infected. For each aphid species, statistically significant differences in responses (*p* < 0.05) between treatments were identified using binomial generalised linear mixed models, fitted with individual experiments (i.e., dots) as a random effect, and using post-hoc tests for pairwise comparisons between treatments (i.e., aphid species), and where lower-case letters are the same, there is no significant difference

Statistical analysis was carried out in RStudio version 4.2.2 [28]. For comparisons testing whether aphids preferred to settle on uninfected plants (Figs. 1 and 2) or to select olfactory cues presented by mock inoculated plants (Fig. 4), the data were analysed using binomial generalised linear mixed models, fitted using the function glmer from the lme4 package [29]. The models were fitted treating replicate experiments as random effects, with a fixed effect for each treatment/time-point of interest. By fitting the models without an overall intercept term, *p*-values against the null hypothesis that the probability of aphids choosing to settle on an uninfected plant was 0.5 could be found via Wald tests of the model’s coefficients for different treatments/time-points of interest. For comparisons testing differences between aphid species in terms of their probabilities of settling on plants (Fig. 3) or for differences in aphid responses to olfactory cues between Mock vs. Mock, Mock vs. Infected and Infected vs. Infected plants for different aphid species (Fig. 5), the data were again analysed using binomial generalised linear mixed models. Again, replicate experiments were modelled as random effects, with a fixed effect for each treatment of interest. The significance of pairwise comparisons between treatments was done using the pairs function in the emmeans package in R [30], with adjustment for multiple comparisons via the Tukey procedure.

## Results

### The establishment of CMV-induced deterrence to settlement by *M. persicae*

It has been shown that by 10–14 days dpi CMV-induced resistance to *M. persicae* is established [12, 13]. This effect appears to drive aphids of this species to settle on mock-inoculated in preference to settling on CMV-infected plants [12]. However, it was unknown how rapidly this effect is manifested after inoculation of *A. thaliana* plants with the virus. Two-way free choice settling assays were carried out at various points after inoculation to understand the establishment of CMV-induced deterrence to aphid settlement on *A. thaliana* plants. Comparison of settling behaviour at 3, 9 and 21 dpi showed that aphids exhibited no preference for either mock-inoculated or CMV-infected plants at 3 dpi. However, aphids preferred settling on mock-inoculated plants by 9 dpi, at which time infection symptoms are visible on uninoculated leaves of infected plants [31] and the aphid preference for mock-inoculated plants was maintained to at least 21 dpi (Fig. 1; File S1).

### Differences in settling preferences of generalist and specialist aphids on CMV-infected and non-infected *A. thaliana* plants

When aphids of the generalist species *M. persicae* are confined on CMV-infected *A. thaliana* plants and unable to emigrate to a mock-inoculated plant, their performance suffers, indicated by decreased growth and fecundity [12–14]. In contrast, aphid growth for *L. erysimi* and *B. brassicae* is unaffected, although *B. brassicae* reproduction is decreased [14]. To determine if these inter-species differences in responses to CMV-induced aphid resistance were reflected in settling behaviour, two-way free choice settling assays were carried out using plants at 14 dpi, i.e., a time-point by which CMV-induced resistance to *M. persicae* would have become established. Whereas *M. persicae* was significantly more likely to settle on mock-inoculated than on CMV-infected plants at either 1 or 24 h post-release, *L. erysimi* behaviour varied between experiments but the overall probability that these aphids would settle on mock-inoculated plants in preference to CMV-infected plants was lower (Fig. 2; File S1; Figure S1). Aphids of *B. brassicae* also showed highly variable settling responses and a decreased likelihood, compared to *M. persicae*, of choosing to settle on mock-inoculated rather than infected plants (Fig. 2; File S1; Figure S1). Further analysis of all data obtained in the settling assays showed that neither of the crucifer-specialist aphids were as likely as *M. persicae* to settle on either infected or non-infected plants within the first hour following release, regardless of the infection status of the plants, i.e., CMV-infected or mock-inoculated (Fig. 3). Although almost all *L. erysimi* and *M. persicae* had settled on plants by 24 h post-release, this was true for just 80% of *B. brassicae* (Fig. 3). These results imply that the crucifer-specialists were less influenced and slower to respond to than *M. persicae* by differences between CMV-infected versus mock-inoculated plants.

### Virus-induced changes in olfactory cues exert the strongest effects on the generalist aphid *M. persicae*

CMV induces changes in the emission of insect-perceivable VOCs by *A. thaliana* plants, probably through the activity of the viral 2b protein [32, 33]. Olfactometry was used to determine if CMV-induced changes in olfactory cues might have contributed to differences observed in aphid settling choices, and if they influence generalist *versus* specialist aphids differentially. Olfactometry with *M. persicae* indicated that more aphids were attracted by VOCs from CMV-infected *A. thaliana* plants than by VOCs from mock-inoculated plants (Fig. 4; File S1; Figure S2). In contrast, neither *B. brassicae* nor *L. erysimi* showed significant preferences for the VOC blends emitted by either mock-inoculated or CMV-infected plants (Fig. 4; File S1; Figure S2).

During olfactometry experiments it was noted that when assays were set up with a choice between mock-inoculated plants only, some aphids did not respond and made no choice. This was most marked with *L. erysimi* and *B. brassicae* (Fig. 5; File S1). A higher proportion of aphids made choices when they were presented with VOCs emitted by CMV-infected plants. This was noted in control assays in which aphids could choose between VOCs from two CMV-infected plants but appeared somewhat stronger when aphids were allowed to choose between VOCs emitted by infected *versus* mock-inoculated plants (Fig. 5; File S1).

## Discussion

CMV induces changes in *A. thaliana* VOC emission that make plants more attractive to *M. persicae* whilst also eliciting changes that make these aphids less likely to settle on infected plants. These alterations are consistent with CMV inducing an ‘attract-and-deter’ type virally modified plant phenotype, which is predicted to increase dispersal of viruliferous aphids from infected to uninfected plants and thereby enhance spread of non-persistently transmitted viruses like CMV [15, 17]. CMV induces this extended phenotype also in squash, affecting the behaviour of both *M. persicae* and another polyphagous aphid, *A. gossypii* [16]. Our initial experiments showed that in *A. thaliana* full establishment of the ‘attract-and-deter’ virally modified plant phenotype does not manifest immediately following infection with CMV and most likely requires systemic infection of the host plants by the virus. An alternative possibility is that CMV might have engendered production of signals in infected tissues that propagated to the rest of the plant ahead of infection. An example of this in *A. thaliana* is systemic signalling via reactive oxygen species induced by cauliflower mosaic virus [34].

Since CMV is among the most important viral pathogens of *A. thaliana* under natural conditions [5], delayed development of virus-induced resistance to aphid settlement may have epidemiological ramifications. Specifically, aphids will not at first be discouraged from settling on CMV-infected plants and may give rise to colonies before the resistance to aphid settlement (the ‘deter’ aspect of the virally induced phenotype) becomes established. Thus, multiple viruliferous aphids may be forced to disperse with the possibility of spreading CMV to multiple neighbouring plants. For this to work, the deterrence to aphid settlement must be greater than the attractiveness of CMV-induced VOC blends. Comparing the results of settling assays with olfactometry assays suggests that for *M. persicae* this is the case. The delayed manifestation of the ‘deter’ component of the CMV-induced ‘attract-and-deter’ plant phenotype could produce a latent period during which CMV-infected plants are effectively non-infectious (at least, with respect to aphid-mediated virus transmission). Other studies with turnip mosaic virus indicated a similar delay in the onset of a ‘repel’ phenotype in *A. thaliana* [35]. Such latent periods in the development of virally induced phenotypes may be relevant to incorporate into future mathematical models of epidemic development.

The effects of CMV on plant-aphid interactions in *A. thaliana* have a far weaker influence on the behaviour of the crucifer-specialist aphids than on *M. persicae*. Although both *B. brassicae* and *L. erysimi* responded to the VOC blend emitted by CMV-infected plants, and possibly more when there was a contrast between VOCs emitted by mock-inoculated plants and CMV-infected plants, aphids of neither specialist species showed any consistent preference between infected plant VOCs and those emitted by mock-inoculated plants (Fig. 5). These two specialist aphids are known to be less affected by CMV-induced resistance to settling (indicated by decreased growth and reproduction of aphid confined on infected plants [14]) than *M. persicae*. This may explain why in the present study the specialists were apparently undeterred from settling on CMV-infected plants. Some differences in their responses to those of *M. persicae* may be explained by the ability of both aphids to tolerate crucifer-specific secondary metabolites such as glucosinolates [36, 37]. Although the results of a previous study indicated that *B. brassicae* (but not *L. erysimi*) may be sensitive to other unidentified CMV-induced compounds [14].

Conceivably, stimulation of non-specific plant-seeking behaviour of the specialist aphids (observed in olfactometry assays when CMV-infected plant VOCs were present) might aid transmission to a limited extent by increasing plant-to-plant aphid migration. However, such a marginal effect could only aid CMV transmission in the case of *L. erysimi*, since *B. brassicae* is not an efficient CMV vector [18]. Taken together, the settling and olfactometric data indicate that while CMV induces an ‘attract-and-deter’ phenotype in *A. thaliana* that influences *M. persicae*, the virus-induced changes affect specialists far less. This suggests that in *A. thaliana* (and by extension, perhaps in other crucifers) CMV-induced changes in host-aphid interactions are most effective at enhancing virus transmission by influencing generalist aphids such as *M. persicae*.

Although the CMV-induced ‘attract-and-deter’ phenotype does not appear to strongly influence specialist aphid interactions with *A. thaliana*, this is not universal. When common bean (*Phaseolus vulgaris*) plants were infected with either CMV or with the potyviruses bean common mosaic virus or bean common mosaic necrosis virus (like CMV, viruses transmitted by aphids in the non-persistent mode) both *M. persicae* and the legume specialist *Aphis fabae* Scopoli responded differentially to infected and non-infected plants. The results of olfactometry, settling assays, and feeding behaviour monitoring were consistent with all three viruses inducing plant phenotypes likely to drive viruliferous aphids (both specialist and non-specialist) away from infected hosts and towards non-infected plants [38, 39]. However, in contrast with our current observations with *A. thaliana*, and those of Mauck et al. [14] with squash, virus-infected bean plants produced VOC blends that repelled aphids [38, 39]. This suggest that only a ‘repel’ phenotype (as defined by Donnelly and colleagues [15]) was induced in bean plants infected with non-persistently transmitted viruses, rather than an ‘attract-and-deter’ phenotype.

Plant VOCs, especially when they occur as blends, provide vital cues for aphids to locate suitable host plants [40]. Previous literature on host-finding by insect herbivores suggests that the process is different between generalists and specialists, especially with respect to accuracy of host plant selection and the time required for an insect to choose between less suitable or more suitable hosts [41–45]. Conclusions have differed on whether specialist or generalist insect herbivores are faster at correct selection of suitable host plants. The ‘neural constraints’ hypothesis proposes that specialist insects will be faster and more accurate because they use a narrower range of plant stimuli to discriminate between potential hosts than generalists [41]. In contrast the ‘sequential cues’ hypothesis proposes that generalists make faster host choice decisions [46], perhaps because they have less to lose than specialists by choosing a slightly less suitable host.

In a recent study with aphids that supports the ‘sequential cues’ hypothesis and appears inconsistent with the older ‘neural constraints’ model, it was found that when generalist aphids (*M. persicae*) were presented with a range of unrelated plant species they discriminated efficiently between hosts and ranked them for greater or lesser suitability for settlement and colony formation [47]. In our own settling and olfactometry assays the generalist aphid *M. persicae* proved to be more ‘decisive’ than either *B. brassicae* or *L. erysimi*. This suggests that, at least for their interactions with *A. thaliana*, aphids appear to conform to the predictions of the ‘sequential cues’ hypothesis.

In summary, we found that the induction of a virally induced host phenotype by CMV occurs gradually as systemic infection progresses, and that in the CMV-*A. thaliana* system the effects of the attract-and-deter phenotype influence generalist aphids more strongly than specialists. Olfactometry suggested that even for the specialist aphids, neither of which showed any preference for CMV-infected over mock-inoculated plant VOCs, one of more components of the blend emitted by infected plants stimulated aphid activity and that activity was further stimulated when there was a contrast between the olfactory stimuli on offer. A possible limitation to this study is that we have used only apterous aphids. Previous work showed that the CMV-induced attract-and-deter phenotype in squash (*Cucurbita pepo* L.) affected apterous and alate *M. persicae* similarly [16]. However, it is conceivable that this may not be true for *A. thaliana* or the specialist aphids used here, and this may be an interesting question for future work.

## Supplementary Information

Supplementary Material 1

Supplementary Material 2

## Data Availability

All datasets generated or analysed during the current study are available in the paper and associated supplementary files.
