# Supplementary material for: Viral manipulation of vector behaviour: cucumber mosaic virus has differential effects on specialist versus generalist aphids infesting Arabidopsis thaliana
Source: Virol J. 2026 Jan 25;23:42. doi: 10.1186/s12985-026-03081-w (PMC12914952; doi:10.1186/s12985-026-03081-w)
Supplement: Supplementary file 2 — Supplementary Material 2 [file 12985_2026_3081_MOESM2_ESM.docx]

**Viral manipulation of vector behaviour: Cucumber mosaic virus has differential effects on specialist versus generalist aphids infesting *Arabidopsis thaliana***

**Hana Azuma, Alex M. Murphy, Nik J. Cunniffe, Arden G. Berlinger and John P. Carr***

Department of Plant Sciences, University of Cambridge, Cambridge CB2 3EA, United Kingdom

* Author for correspondence; jpc1005@cam.ac.uk

**Supplementary Figures**

| 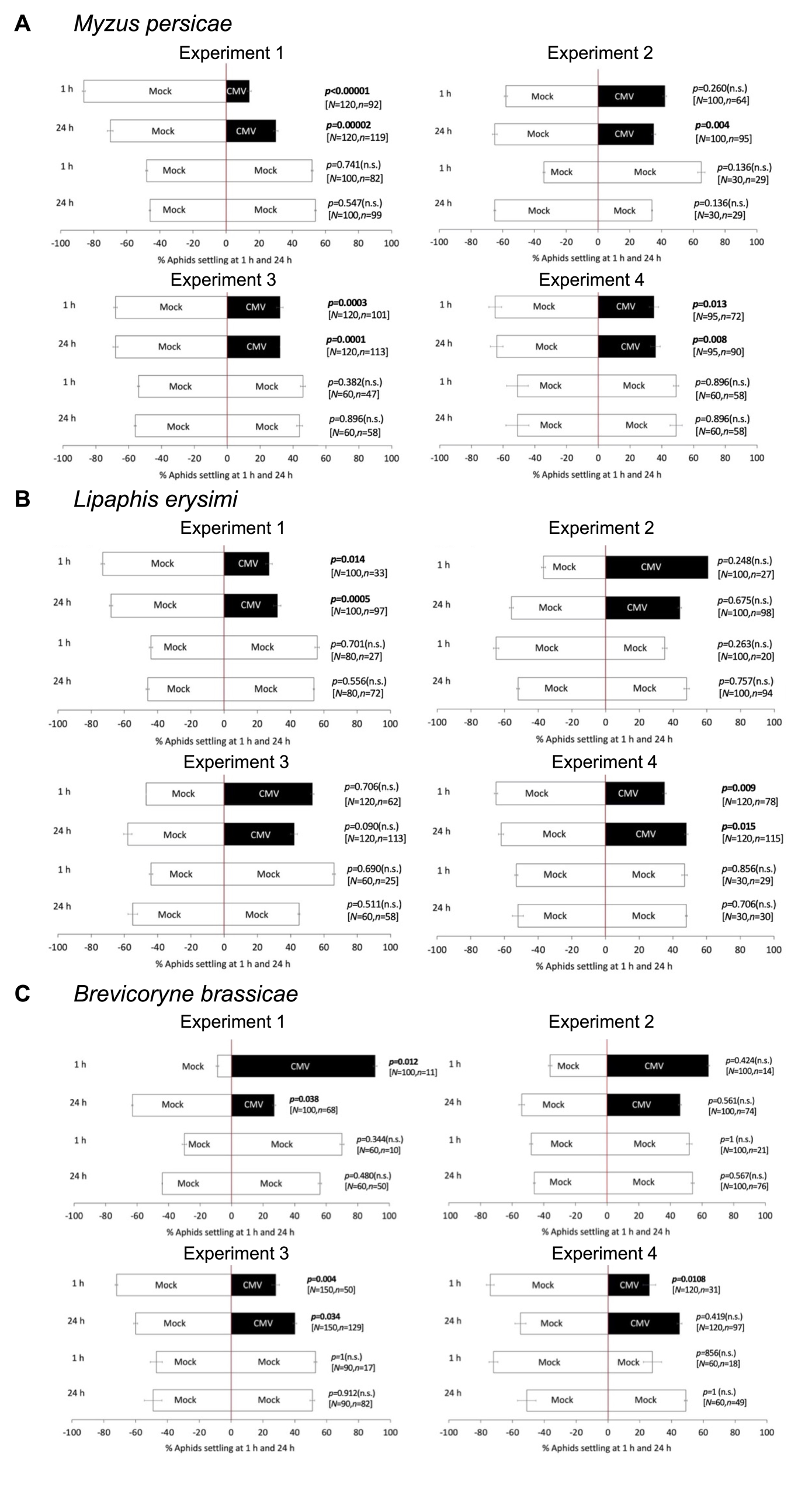 |
| --- |

**Figure S1. Settling assays.** Four free choice settling experiments were carried out for each aphid: *Myzus persicae* (A), *Lipaphis erysimi* (B), and *Brevicoryne brassicae*. Aphids were allowed to choose between the treatments shown, i.e., mock-inoculated plants (Mock) versus plants infected with Fny-CMV (CMV). Aphids settled on plants were counted (n) at 1 and 24h after release of N aphids. Differences in settlement were assessed for statistical significance using a binomial test with ‘n.s.’ indicating non-significant differences.

|  |
| --- |

**Figure S2. Olfactometry assays.** Experiments were carried out for *Myzus persicae* (A), *Lipaphis erysimi* (B), and *Brevicoryne brasssicae* to determine preferences for volatile organic compounds emitted by mock-inoculated plants (Mock) versus plants infected with Fny-CMV (CMV). Aphids making choices by 24h post-release were counted (n) and N is the total number of aphids assayed. Differences in settlement were assessed for statistical significance using a binomial test with ‘n.s.’ indicating non-significant differences.
